# Integrating preliminary test and Stein-type techniques to improve estimation in the time-dependent Cox model

**DOI:** 10.1371/journal.pone.0345123

**Published:** 2026-06-08

**Authors:** Rohollah Ramezani, Mohammad Reza Rabiei, Mohammad Arashi

**Affiliations:** 1 Department of Statistics, Faculty of Mathematical Sciences, Shahrood University of Technology, Shahrood, Iran; 2 Department of Statistics, Faculty of Mathematical Sciences, Ferdowsi University of Mashhad, Mashhad, Iran; University of Mosul, IRAQ

## Abstract

While shrinkage and preliminary test estimation have long been studied in linear and static Cox models, their theoretical integration within models featuring time-dependent covariates has remained unresolved due to the evolving risk set and nonhomogeneous information accumulation inherent in such data. In this study, we develop a unified framework for shrinkage estimation in the time-dependent Cox proportional hazards model, by extending the classical Stein-type theory to a dynamic semiparametric survival setting. Our theoretical analyses reveal that the positive-rule Stein estimator preserves unbiasedness under valid restrictions while adaptively attenuating variance inflation when the restriction is approximately correct, striking a principled balance between efficiency and robustness. A comprehensive Monte Carlo simulation study and an empirical application to the Mayo Clinic primary biliary cirrhosis dataset substantiate the theoretical advantages, demonstrating that the superior estimation strategy achieves substantial efficiency gains relative to both unrestricted and penalized estimators such as adaptive LASSO.

## Introduction

Since the pioneering work of Cox (1972) [1], the proportional hazards model has become a foundational tool for survival analysis. Its semiparametric formulation enables flexible assessment of covariate effects on event times, yet the classical model assumes time-invariant effects—an assumption often violated in biomedical studies where risk factors evolve dynamically. Time-dependent extensions of the Cox model—originally formalized via the counting-process and martingale framework by Andersen and Gill (1982) [2] and subsequently advanced by Fleming and Harrington (2005) [3] and Andersen et al. (1995) [4]—provide a rigorous theoretical foundation for estimation and inference in the presence of dynamic covariates.

Although the partial likelihood estimator is consistent and asymptotically normal under standard regularity conditions [2], its finite-sample efficiency may deteriorate in the presence of weak signals, multicollinearity, or when credible prior information about linear constraints is available. Shrinkage methods offer a principled strategy for improving efficiency by introducing controlled bias. Originating from the seminal contributions of Stein (1956) [5] and James and Stein (1992) [6], this idea has stimulated extensive research across linear, ridge-type, robust, and high-dimensional settings [7–11]. A modern perspective on shrinkage and its empirical Bayes interpretation is given by Efron (2024) [12].

Despite these advances, shrinkage estimation for time-dependent Cox models remains comparatively underdeveloped. Existing work has largely focused on time-independent covariates, where risk sets and information accumulation are more tractable. In contrast, time-dependent covariates introduce evolving risk structures, nonhomogeneous information processes, and more intricate local asymptotic behavior.

While asymptotic properties under local alternatives have been studied in classical linear models and Cox models with time-independent covariates [13], existing results typically do not characterize the joint behavior of restricted and unrestricted estimators in the presence of time-dependent covariates. Moreover, the integration of such asymptotic frameworks with Stein-type shrinkage estimation remains limited. These considerations highlight the need for a unified treatment of shrinkage estimation in this semiparametric setting.

Parallel developments in variable selection for Cox models—ranging from classical stepwise procedures [13] to modern penalized likelihood methods such as LASSO [14], adaptive LASSO, Elastic Net (ENET), SCAD [15], and group LASSO [16]—have demonstrated excellent empirical performance, especially in correlated or moderately high-dimensional data. However, penalized estimators do not naturally incorporate approximate linear restrictions, nor do they exhibit the adaptive risk-balancing behavior characteristic of Stein-type methods.

This paper addresses this gap by developing a unified shrinkage framework for the time-dependent Cox proportional hazards model. We introduce three improved estimators—the Preliminary Test Estimator (PTE), the Stein-type Estimator (SE), and the Positive-Rule Stein Estimator (PSE)—and derive their asymptotic distributions under local alternatives using counting-process theory. Closed-form expressions for asymptotic bias, risk, and mean squared error are obtained, clarifying the conditions under which shrinkage provides meaningful efficiency gains relative to unrestricted partial likelihood and penalized estimators.

The theoretical results are complemented with extensive Monte Carlo experiments and a real-data analysis of the Mayo Clinic PBC cohort. Across diverse scenarios, the PSE achieves the most favorable bias–variance trade-off and often outperforms both the unrestricted estimator and modern penalized approaches such as adaptive LASSO.

The remainder of the paper is structured as follows. *The Time-Dependent Cox Model* introduces the time-dependent Cox framework and outlines the associated asymptotic properties. *Shrinkage Estimation under the Time-Dependent Cox Model* presents the proposed shrinkage estimators and establishes their theoretical results. *Simulation Study* reports Monte Carlo evidence evaluating finite-sample performance, whereas *Application to the Mayo Clinic PBC Data* demonstrates the practical applicability of the proposed methodology using real-world data. Finally, *Conclusions* summarizes the main findings and discusses potential directions for future research.

## The time-dependent Cox model

Let *T*_*i*_ and *C*_*i*_ denote the event and censoring times for subject *i*, with observed time $t_{i}=\min(T_{i},C_{i})$ and indicator $\delta_{i}=I(T_{i}\leq C_{i})$, i=1,…,n. The covariate process is denoted by ${𝐗}_{i}(t)=(X_{i1}(t),\ldots ,X_{ip}(t){)}^{⊤}$, allowing the effect of predictors to vary over time. Let *Y*_*i*_(*t*) denote the at-risk indicator process.

The time-dependent Cox model specifies the hazard function as


$$\begin{array}{c} h_{i}(t|{𝐗}_{i}(t))=h_{0}(t)\exp(\beta^{⊤}{𝐗}_{i}(t)), \end{array}$$ (1)

where *h*_0_(*t*) is an unspecified baseline hazard and $\beta \in ℬ\subset {\mathbb{R}}^{p}$ is a vector of regression coefficients, with ℬ an open parameter space.

To establish the asymptotic properties of the proposed estimators, we adopt regularity conditions analogous to those of Andersen and Gill (1982) [2], rewritten in terms of the notation used in this paper. Throughout, ${𝐗}^{⊗k}$ denotes the *k*-fold tensor product.

(C1) **Finite cumulative baseline hazard.** The baseline hazard function satisfies
  $$\begin{array}{c} \int_{0}^{\tau }h_{0}(t)\;dt<\infty , \end{array}$$
  for some finite τ>0.
(C2) **Stability of risk processes.** Define
  $$\begin{array}{c} S_{n}^{\left(k\right)}(\beta ,t)=\frac{1}{n}\sum_{i=1}^{n}Y_{i}(t){𝐗}_{i}(t{)}^{⊗k}\exp\{\beta^{⊤}{𝐗}_{i}(t)\},\;k=0,1,2. \end{array}$$
  There exist deterministic limit functions $s^{\left(k\right)}(\beta ,t)$ such that
  $$\begin{array}{c} \underset{t\in [0,\tau ]}{\sup}\underset{\beta \in ℬ}{\sup}\left\|S_{n}^{\left(k\right)}(\beta ,t)-s^{\left(k\right)}(\beta ,t)\right\|\overset{P\;}{\to }0, \end{array}$$
  for *k* = 0,1,2.
(C3) **Lindeberg-type condition.** For some δ>0,
  $$\begin{array}{c} \frac{1}{n}\sum_{i=1}^{n}𝔼\left[\|{𝐗}_{i}(t){\|}^{2}1\{\|{𝐗}_{i}(t)\|>\delta \sqrt{n}\}\right]\to 0. \end{array}$$
(C4) **Regularity and boundedness.** The limit functions $s^{\left(k\right)}(\beta ,t)$ are continuous in β uniformly in t∈[0,τ], and bounded. Moreover, $s^{\left(0\right)}(\beta ,t)$ is bounded away from zero.
  Define
  $$\begin{array}{c} 𝐞(\beta ,t)=\frac{s^{\left(1\right)}(\beta ,t)}{s^{\left(0\right)}(\beta ,t)},\;𝐯(\beta ,t)=\frac{s^{\left(2\right)}(\beta ,t)}{s^{\left(0\right)}(\beta ,t)}-𝐞(\beta ,t{)}^{⊗2}. \end{array}$$
  Then the matrix
  $$\begin{array}{c} \Sigma (\beta )=\int_{0}^{\tau }𝐯(\beta ,t)s^{\left(0\right)}(\beta ,t)h_{0}(t)\;dt \end{array}$$
  is positive definite.

### Partial likelihood

Estimation of β is based on the partial likelihood [2,4]:


$$\begin{array}{c} L(\beta )=\prod_{i=1}^{n}{\left[\frac{\exp(\beta^{⊤}{𝐗}_{i}(t_{i}))}{\sum_{j\in R(t_{i})}\exp(\beta^{⊤}{𝐗}_{j}(t_{i}))}\right]}^{\delta_{i}}, \end{array}$$ (2)

where $R(t_{i})=\{j:t_{j}\geq t_{i}\}$ is the risk set at time *t*_*i*_. Maximizing the full likelihood with respect to the baseline cumulative hazard *H*_0_(*t*) shows that the profile likelihood $L_{p}(\beta )$ is proportional to (2) [17].

In the presence of tied event times, Efron’s approximation [18] provides an accurate and computationally efficient modification:


$$\begin{array}{c} L^{Efr}(\beta )=\prod_{i=1}^{n}\frac{\prod_{l\in D_{t_{i}}}\exp(\beta^{⊤}{𝐗}_{l}(t_{i}))}{\prod_{k=1}^{d_{i}}\left(\sum_{j\in R(t_{i})}\exp(\beta^{⊤}{𝐗}_{j}(t_{i}))-\frac{k-1}{d_{i}}\sum_{j\in D_{t_{i}}}\exp(\beta^{⊤}{𝐗}_{j}(t_{i}))\right)}, \end{array}$$ (3)

where $D_{t_{i}}$ is the set of individuals failing at time *t*_*i*_ and $d_{i}=|D_{t_{i}}|$.

### Semiparametric estimation

Given the conditional hazard function in (1), inference for β is based on the partial likelihood with Efron’s correction for tied event times [3,18,19]. Let $\ell^{Efr}(\beta )$ denote the corresponding log–partial likelihood, with


$$\begin{array}{c} A_{ik}=\sum_{j\in R(t_{i})}e^{\beta^{⊤}{𝐗}_{j}(t_{i})}-\frac{k-1}{d_{i}}\sum_{j\in D_{i}}e^{\beta^{⊤}{𝐗}_{j}(t_{i})},\\ B_{ik}=\sum_{j\in R(t_{i})}{𝐗}_{j}(t_{i})e^{\beta^{⊤}{𝐗}_{j}(t_{i})}-\frac{k-1}{d_{i}}\sum_{j\in D_{i}}{𝐗}_{j}(t_{i})e^{\beta^{⊤}{𝐗}_{j}(t_{i})}. \end{array}$$

The score function and observed information matrix are then


$$\begin{array}{c} U_{n}(\beta )=\sum_{i=1}^{n}\left[\sum_{l\in D_{i}}{𝐗}_{l}(t_{i})-\sum_{k=1}^{d_{i}}\frac{B_{ik}}{A_{ik}}\right], \end{array}$$

$$\begin{array}{c} I_{n}(\beta )=-\frac{\partial U_{n}(\beta )}{\partial \beta^{⊤}}=\sum_{i=1}^{n}\left[\sum_{k=1}^{d_{i}}\frac{A_{ik}\frac{\partial B_{ik}}{\partial \beta^{⊤}}-B_{ik}\frac{\partial A_{ik}}{\partial \beta^{⊤}}}{A_{ik}^{2}}\right]. \end{array}$$

The unrestricted estimator (UE) is the maximizer of the partial likelihood, ${\hat{\beta }}_{n}^{pl}=\text{arg}\max_{\beta }\;\ell^{Efr}(\beta ),$ computed via the Newton–Raphson/Fisher–scoring iteration $\beta^{k+1}=\beta^{k}-I_{n}^{-1}(\beta^{k})\;U_{n}(\beta^{k}).$ The asymptotic theory of [2] ensures that, under regularity conditions (C1–C4), ${\hat{\beta }}_{n}^{pl}\overset{P\;}{\to }\beta ,\;\sqrt{n}({\hat{\beta }}_{n}^{pl}-\beta )\overset{D\;}{\to }{𝒩}_{p}\;\left(0,\Sigma (\beta {)}^{-1}\right)$ and that the observed information satisfies $n^{-1}I_{n}({\hat{\beta }}_{n}^{pl})\overset{P\;}{\to }\Sigma (\beta )$.

However, in many applications, partial prior information on the parameter may be available in the form of linear constraints $H_{0}:\;H\beta =h,$ where ***H*** is a *q* × *p* full–rank matrix. When *H*_0_ holds, the restricted estimator (RE) is obtained by projecting the UE onto the constrained subspace:


$$\begin{array}{c} {\hat{\beta }}_{n}^{R}={\hat{\beta }}_{n}^{pl}-I_{n}^{-1}({\hat{\beta }}_{n}^{pl})\;H^{⊤}(HI_{n}^{-1}({\hat{\beta }}_{n}^{pl})H^{⊤}{)}^{-1}(H{\hat{\beta }}_{n}^{pl}-h). \end{array}$$

Under *H*_0_, and using the convergence $n^{-1}I_{n}({\hat{\beta }}_{n}^{pl})\overset{P\;}{\to }\Sigma (\beta )$, it follows from Slutsky’s theorem that


$$\begin{array}{c} \sqrt{n}({\hat{\beta }}_{n}^{R}-\beta )\overset{D\;}{\to }{𝒩}_{p}\;\left(0,\;A\right), \end{array}$$

where


$$\begin{array}{c} A=\Sigma (\beta {)}^{-1}-\Sigma (\beta {)}^{-1}H^{⊤}(H\Sigma (\beta {)}^{-1}H^{⊤}{)}^{-1}H\Sigma (\beta {)}^{-1}. \end{array}$$

This unified framework provides the basis for constructing the shrinkage and pretest estimators developed in following Section.

## Shrinkage estimation under the time-dependent Cox model

In semiparametric survival models, incorporating external information in the form of linear constraints $H_{0}:H\beta =h$ can yield substantial efficiency gains when the restriction is valid. The UE and RE estimators therefore represent two extremes: full reliance on the data versus full reliance on the constraint. Since the validity of *H*_0_ is typically uncertain, methods that adaptively interpolate between these extremes are of both practical and theoretical interest.

To evaluate the plausibility of *H*_0_, we employ the Wald statistic


$$\begin{array}{c} {𝒲}_{n}=n(H{\hat{\beta }}_{n}^{pl}-h{)}^{⊤}(HI_{n}^{-1}({\hat{\beta }}_{n}^{pl})H^{⊤}{)}^{-1}(H{\hat{\beta }}_{n}^{pl}-h), \end{array}$$

which is asymptotically $\chi_{q}^{2}$ under *H*_0_ and noncentral $\chi_{q}^{2}$ under *H*_*A*_. This leads to the classical preliminary test estimator,


$$\begin{array}{c} {\hat{\beta }}_{n}^{PTE}=I({𝒲}_{n}<\chi_{q,\alpha }^{2}){\hat{\beta }}_{n}^{R}+\{1-I({𝒲}_{n}<\chi_{q,\alpha }^{2})\}{\hat{\beta }}_{n}^{pl}, \end{array}$$

which, however, inherits the discontinuity and significance–level dependence typical of pretest procedures.

A continuous alternative is provided by a James–Stein shrinkage estimator,


$$\begin{array}{c} {\hat{\beta }}_{n}^{S}={\hat{\beta }}_{n}^{pl}-d\;({\hat{\beta }}_{n}^{pl}-{\hat{\beta }}_{n}^{R}){𝒲}_{n}^{-1},\;d\geq 0, \end{array}$$

which shrinks the UE toward the RE with data‐adaptive intensity. Since ${\hat{\beta }}_{n}^{S}$ may overshoot the restricted subspace, we employ the positive–rule modification


$$\begin{array}{c} {\hat{\beta }}_{n}^{S+}={\hat{\beta }}_{n}^{R}+\{1-d{𝒲}_{n}^{-1}\}I({𝒲}_{n}>d)({\hat{\beta }}_{n}^{pl}-{\hat{\beta }}_{n}^{R}), \end{array}$$

which enforces a nonnegative shrinkage factor and yields improved stability.

These estimators form a coherent class of shrinkage procedures that continuously blend restricted and unrestricted inference, enabling principled incorporation of uncertain prior information in the time–dependent Cox proportional hazards model.

### Theoretical characteristics

In this section, we introduce the asymptotic distributional bias (ADB), quadratic bias (ADQB), MSE-matrices (ADMSE), and quadratic risks (ADQR) associated with estimators of the parameter vector β. To this end, let $\beta_{n}^{*}$ denote a general consistent estimator of β, and consider a positive semi-definite weight matrix ***W*** along with the following quadratic loss function:


$$\begin{array}{c} L(\beta_{n}^{*},\beta )=n(\beta_{n}^{*}-\beta {)}^{⊤}W(\beta_{n}^{*}-\beta )=\text{tr}\left[W\{n(\beta_{n}^{*}-\beta )(\beta_{n}^{*}-\beta {)}^{⊤}\}\right]. \end{array}$$ (4)

Let the asymptotic distribution matrix (ADM) be


$$\begin{array}{c} M(\beta_{n}^{*})=\lim_{n\to \infty }E\{n(\beta_{n}^{*}-\beta )(\beta_{n}^{*}-\beta {)}^{⊤}\}, \end{array}$$

Then the A’DQR of $\beta_{n}^{*}$ is given by $R(\beta_{n}^{*};W)=\text{tr}[WM(\beta_{n}^{*})]$.

Under fixed alternative hypotheses $K_{\pi }:H\beta =h+\pi$, it follows that $\sqrt{n}\;I_{n}^{1/2}({\hat{\beta }}_{n}^{pl})(\beta_{n}^{*}-\beta )$ converges in distribution to the same limiting distribution as $\sqrt{n}\;\Sigma^{1/2}(\beta )({\hat{\beta }}_{n}^{pl}-\beta ),$ as n→∞. This follows from the consistency of $n^{-1}I_{n}({\hat{\beta }}_{n}^{pl})$ for $\Sigma^{-1}(\beta )$ [2,20] together with Slutsky’s theorem.

Then, To achieve a meaningful asymptotic distribution of $\sqrt{n}I_{n}^{1/2}({\hat{\beta }}_{n}^{pl})(\beta_{n}^{*}-\beta )$, we look at the class of local alternatives, {*K*_(*n*)_} is given by $K_{\left(n\right)}:H\beta =h+n^{-1/2}\pi$. Let the asymptotic cumulative distribution function (cdf) of $\sqrt{n}I_{n}^{1/2}({\hat{\beta }}_{n}^{pl})(\beta_{n}^{*}-\beta )$ under {*K*_(*n*)_} be


$$\begin{array}{c} G_{p}(x)=\lim_{n\to \infty }P_{K_{\left(n\right)}}\{\sqrt{n}I_{n}^{1/2}({\hat{\beta }}_{n}^{pl})(\beta_{n}^{*}-\beta )\leq x\}. \end{array}$$ (5)

If the asymptotic cdf exists, then the ADB and the ADQB are given by


$$\begin{array}{c} b(\beta_{n}^{*})=\lim_{n\to \infty }E\{\sqrt{n}I_{n}^{1/2}({\hat{\beta }}_{n}^{pl})(\beta_{n}^{*}-\beta ))\}=\int xdG_{p}(x), \end{array}$$ (6)

and $B(\beta_{n}^{*})=[b(\beta_{n}^{*}){]}^{⊤}\Sigma (\beta )[b(\beta_{n}^{*})]$, respectively, where $\Sigma^{-1}(\beta )$ is the MSE-matrix of ${\hat{\beta }}_{n}^{pl}$ as n→∞. Defining $M(\beta_{n}^{*})=\int xx^{\prime }dG_{p}(x)$, we have the weighted risk of $\beta_{n}^{*}$ given by $R(\beta_{n}^{*})=\text{tr}[WM(\beta_{n}^{*})]$.

#### Asymptotics under the fixed alternatives.

We now turn to the asymptotic behavior of the Wald statistic ${𝒲}_{n}$ when the null hypothesis $H_{0}:H\beta =h$ is violated. In particular, we investigate its limiting distribution under fixed alternatives of the form $K_{\pi }:H\beta =h+\pi$, where π is a fixed vector in ${\mathbb{R}}^{q}$. Then


$$\begin{array}{cc} \sqrt{n}I_{n}^{1/2}({\hat{\beta }}_{n}^{pl})(H{\hat{\beta }}_{n}^{pl}-h) & =\sqrt{n}I_{n}^{1/2}({\hat{\beta }}_{n}^{pl})\{H({\hat{\beta }}_{n}^{pl}-\beta )+(H\beta -h)\}\\ & =\sqrt{n}I_{n}^{1/2}({\hat{\beta }}_{n}^{pl})H({\hat{\beta }}_{n}^{pl}-\beta )+\sqrt{n}I_{n}^{1/2}({\hat{\beta }}_{n}^{pl})\pi , \end{array}$$

and the statistic ${𝒲}_{n}$ can be written as


$$\begin{array}{cc} {𝒲}_{n} & =n({\hat{\beta }}_{n}^{pl}-\beta {)}^{⊤}H^{⊤}(HI_{n}^{-1}({\hat{\beta }}_{n}^{pl})H^{⊤}{)}^{-1}H({\hat{\beta }}_{n}^{pl}-\beta )\\ & \;+n\pi^{⊤}(HI_{n}^{-1}({\hat{\beta }}_{n}^{pl})H^{⊤}{)}^{-1}\pi +2n({\hat{\beta }}_{n}^{pl}-\beta {)}^{⊤}H^{⊤}(HI_{n}^{-1}({\hat{\beta }}_{n}^{pl})H^{⊤}{)}^{-1}\pi . \end{array}$$ (7)

Under the assumed regularity conditions C1–C4 and invoking Theorem 4.2 of [2], as n→∞, we obtain


$$\begin{array}{c} \sqrt{n}\;I_{n}^{1/2}({\hat{\beta }}_{n}^{pl})(H{\hat{\beta }}_{n}^{pl}-h)=\sqrt{n}\;I_{n}^{1/2}({\hat{\beta }}_{n}^{pl})\pi +{𝐙}_{n}+o_{P}(1), \end{array}$$ (8)

$$\begin{array}{c} {𝐙}_{n}=\sqrt{n}I_{n}^{1/2}({\hat{\beta }}_{n}^{pl})H({\hat{\beta }}_{n}^{pl}-\beta )\overset{d}{⟶}{𝒩}_{q}\;\left(0,(H\Sigma^{-1}(\beta )H^{⊤}{)}^{-1}\right), \end{array}$$

and


$$\begin{array}{c} n\pi^{⊤}(HI_{n}^{-1}({\hat{\beta }}_{n}^{pl})H^{⊤}{)}^{-1}\pi \overset{P\;}{\to }\infty \;\text{as }n\to \infty . \end{array}$$

Moreover, the cross term satisfies


$$\begin{array}{c} 2n({\hat{\beta }}_{n}^{pl}-\beta {)}^{⊤}H^{⊤}(HI_{n}^{-1}({\hat{\beta }}_{n}^{pl})H^{⊤}{)}^{-1}\pi =o_{P}(\sqrt{n}), \end{array}$$

and hence diverges in probability as n→∞. Therefore, it follows that


$$\begin{array}{c} {𝒲}_{n}\overset{P\;}{\to }\infty \;\text{as }n\to \infty . \end{array}$$

Consequently, for any fixed *x* > 0,


$$\begin{array}{c} P\{{𝒲}_{n}>x\}\to 1\;\text{as }n\to \infty , \end{array}$$

under the fixed alternatives $K_{\pi }:H\beta =h+\pi$. Then we have the following theorem:

**Theorem 1.** *Under the regularity conditions C1–C4, and under the fixed alternatives* $K_{\pi }:H\beta =h+\pi$*, as* n→∞*, we have*


$$\begin{array}{c} \text{(i)}\;\sqrt{n}I_{n}^{1/2}({\hat{\beta }}_{n}^{pl})({\hat{\beta }}_{n}^{PTE}-{\hat{\beta }}_{n}^{pl})=\sqrt{n}\Sigma^{1/2}(\beta )({\hat{\beta }}_{n}^{pl}-\beta )+o_{P}(1),\\ \text{(ii)}\;\sqrt{n}I_{n}^{1/2}({\hat{\beta }}_{n}^{pl})({\hat{\beta }}_{n}^{S}-{\hat{\beta }}_{n}^{pl})=\sqrt{n}\Sigma^{1/2}(\beta )({\hat{\beta }}_{n}^{pl}-\beta )+o_{P}(1),\\ \text{(iii)}\;\sqrt{n}I_{n}^{1/2}({\hat{\beta }}_{n}^{pl})({\hat{\beta }}_{n}^{S+}-{\hat{\beta }}_{n}^{pl})=\sqrt{n}\Sigma^{1/2}(\beta )({\hat{\beta }}_{n}^{pl}-\beta )+o_{P}(1). \end{array}$$ (9)

The proof of Theorem 1 is provided in the Appendix.

Theorem 1 shows that the asymptotic distribution of $\sqrt{n}({\hat{\beta }}_{n}^{PTE}-\beta )$, $\sqrt{n}({\hat{\beta }}_{n}^{S}-\beta )$, and $\sqrt{n}({\hat{\beta }}_{n}^{S+}-\beta )$ is given by ${𝒩}_{p}(0,\Sigma^{-1}(\beta ))$, and the ADB,ADQB, ADMSE, and ADQR of the estimators ${\hat{\beta }}_{n}^{PTE}$, ${\hat{\beta }}_{n}^{S}$, and ${\hat{\beta }}_{n}^{S+}$ are all equal and are given by


$$\begin{array}{c} b_{1}({\hat{\beta }}_{n}^{pl})=b_{3}({\hat{\beta }}_{n}^{PTE})=b_{4}({\hat{\beta }}_{n}^{S})=b_{5}({\hat{\beta }}_{m}^{S+})=0,\\ M_{3}({\hat{\beta }}_{n}^{PTE})=M_{4}({\hat{\beta }}_{n}^{S})=M_{5}({\hat{\beta }}_{n}^{S+})=\Sigma^{-1}(\beta ), \end{array}$$

$$\begin{array}{c} R_{1}({\hat{\beta }}_{n}^{pl};𝐖)=R_{3}({\hat{\beta }}_{n}^{PTE};𝐖)=R_{4}({\hat{\beta }}_{n}^{S};𝐖)=R_{5}({\hat{\beta }}_{n}^{S+};𝐖)=\text{tr}(𝐖\Sigma^{-1}(\beta )) \end{array}$$ (10)

while $n\|{\hat{\beta }}_{n}^{pl}-{\hat{\beta }}_{n}^{R}{\|}_{\Sigma^{-1}(\beta )}^{2}={𝒲}_{n}\overset{p\;}{\to }\infty \;\text{as}\;n\to \infty ;$ and $E({𝒲}_{n})\to \infty$ as n→∞. Thus, the asymptotic distributions of $\sqrt{n}I_{n}^{1/2}({\hat{\beta }}_{n}^{pl})({\hat{\beta }}_{n}^{R}-\beta )$ and $\sqrt{n}I_{n}^{1/2}(\beta_{n}^{pl})({\hat{\beta }}_{n}^{pl}-\beta )$ are not equivalent as n→∞. Now,


$$\begin{array}{cc} \sqrt{n}I_{n}^{1/2}({\hat{\beta }}_{n}^{pl})({\hat{\beta }}_{n}^{R}-\beta ) & =\sqrt{n}I_{n}^{1/2}({\hat{\beta }}_{n}^{pl})({\hat{\beta }}_{n}^{pl}-\beta )\\ & \;-\sqrt{n}I_{n}^{-1/2}({\hat{\beta }}_{n}^{pl})H^{⊤}(HI^{-1}({\hat{\beta }}_{n}^{pl})H^{⊤}{)}^{-1}(H({\hat{\beta }}_{n}^{pl}-\beta )+\pi ) \end{array}$$ (11)

(proof in the Appendix) implies that the asymptotic distribution of $\sqrt{n}I_{n}^{1/2}({\hat{\beta }}_{n}^{pl})({\hat{\beta }}_{n}^{R}-\beta )$ is degenerate under the fixed alternative $K_{\pi }$.

#### Asymptotics under the local alternatives.

To obtain meaningful asymptotic distributions of the various estimators and the test-statistics, ${𝒲}_{n}$ we consider the following theorem: **Theorem 2.** *Under* $K_{\left(n\right)}:H\beta =h+n^{-1/2}\pi$ *and under the regularity conditions C1–C4, the following result holds as* n→∞*:*


$$\begin{array}{cc} \text{(i)}\; & {𝐕}_{n}^{\left(1\right)}=\sqrt{n}({\hat{\beta }}_{n}^{pl}-\beta )\sim {𝒩}_{p}(0,\Sigma^{-1}(\beta )),\\ \text{(ii)}\; & {𝐕}_{n}^{\left(2\right)}=\sqrt{n}({\hat{\beta }}_{n}^{R}-\beta )\sim {𝒩}_{p}(-\delta ,𝐀),\text{ where }\delta =\Sigma^{-1}(\beta )H^{⊤}(H\Sigma^{-1}(\beta )H^{⊤}{)}^{-1}\pi ,\\ & \;\text{and }𝐀=\Sigma^{-1}(\beta )-\Sigma^{-1}(\beta )H^{⊤}(H\Sigma^{-1}(\beta )H^{⊤}{)}^{-1}H\Sigma^{-1}(\beta ),\\ \text{(iii)}\; & {𝐕}_{n}^{\left(3\right)}=\sqrt{n}({\hat{\beta }}_{n}^{pl}-{\hat{\beta }}_{n}^{R})\sim {𝒩}_{p}(\delta ,\Sigma^{-1}(\beta )-𝐀),\\ \text{(iv)}\; & [\begin{array}{c} {𝐕}_{n}^{\left(1\right)}\\ {𝐕}_{n}^{\left(3\right)} \end{array}]\sim {𝒩}_{2p}\left((\begin{array}{c} 0\\ \delta \end{array}),(\begin{array}{cc} \Sigma^{-1}(\beta ) & \Sigma^{-1}(\beta )-𝐀\\ \Sigma^{-1}(\beta )-𝐀 & \Sigma^{-1}(\beta )-𝐀 \end{array})\right),\\ \text{(v)}\; & [\begin{array}{c} {𝐕}_{n}^{\left(2\right)}\\ {𝐕}_{n}^{\left(3\right)} \end{array}]\sim {𝒩}_{2p}\left((\begin{array}{c} -\delta \\ \delta \end{array}),(\begin{array}{cc} 𝐀 & 0\\ 0 & \Sigma^{-1}(\beta )-𝐀 \end{array})\right),\\ \text{(vi)}\; & \lim_{n\to \infty }P({𝒲}_{n}\leq x)=H_{q}(x;\Delta^{2}),\\ \text{(vii)}\; & \lim_{n\to \infty }P\{\sqrt{n}({\hat{\beta }}_{n}^{PTE}-\beta )\leq x\}=H_{q}(\chi_{q}^{2}(\alpha );\Delta^{2})\Phi_{p}(x+\delta ,0,𝐀)\\ & \;+\int_{E(\delta )}\Phi_{p}(x+𝐙,0,(\Sigma^{-1}(\beta )-𝐀))\;d\Phi_{p}(𝐙,0,(𝐇\Sigma^{-1}(\beta )H^{⊤})). \end{array}$$ (12)

*where* $E(\delta )=\{𝐙:(𝐙+\delta {)}^{⊤}(𝐇\Sigma^{-1}(\beta )H^{⊤}{)}^{-1}(𝐙+\delta )\geq \chi_{q}^{2}(\alpha )\}$*, and* $\Phi_{p}(\cdot ;\mu ,V)$ *is the cdf of a p-variate normal distribution with mean* μ *and covariance matrix* ***V****, and* $H_{v}(\cdot ;\Delta^{2})$ *is the cdf of a noncentral chi-square distribution with v d.f. and noncentrality parameter* $\Delta^{2}$.

*It is difficult to obtain the asymptotic distributions of* $\sqrt{n}({\hat{\beta }}_{n}^{S}-\beta )$ *and* $\sqrt{n}({\hat{\beta }}_{n}^{S+}-\beta )$*, but we can obtain an asymptotic representation of these estimators under {K*_*(n)*_*} to facilitate the computation of ADB, ADQB, ADMSE and ADQR of these estimators. Hence, we have under {K*_*(n)*_*}*


$$\begin{array}{cc} \text{(viii)}\; & \sqrt{n}({\hat{\beta }}_{n}^{S}-\beta )\overset{d}{=}𝐙-k\{\frac{\Sigma^{-1}(\beta )H^{⊤}(H\Sigma^{-1}(\beta )H^{⊤}{)}^{-1}(𝐇𝐙+\pi )}{\left(𝐇𝐙+\pi {)}^{⊤}(𝐇\Sigma^{-1}(\beta )H^{⊤}{)}^{-1}(𝐇𝐙+\pi \right)}\},\\ \text{(ix)}\; & \sqrt{n}({\hat{\beta }}_{n}^{S+}-\beta )\overset{d}{=}𝐙-k\{\frac{\Sigma^{-1}(\beta )H^{⊤}(H\Sigma^{-1}(\beta )H^{⊤}{)}^{-1}(𝐇𝐙+\pi )}{\left(𝐇𝐙+\pi {)}^{⊤}(𝐇\Sigma^{-1}(\beta )H^{⊤}{)}^{-1}(𝐇𝐙+\pi \right)}\}\\ & \;+\Sigma^{-1}(\beta )H^{⊤}(H\Sigma^{-1}(\beta )H^{⊤}{)}^{-1}(𝐇𝐙+\pi )\\ & \;\times [1-\frac{k}{\left(𝐇𝐙+\pi {)}^{⊤}(𝐇\Sigma^{-1}(\beta )H^{⊤}{)}^{-1}(𝐇𝐙+\pi \right)}]\\ & \;\times I\{(𝐇𝐙+\pi {)}^{⊤}(𝐇\Sigma^{-1}(\beta )H^{⊤}{)}^{-1}(𝐇𝐙+\pi )<k\}, \end{array}$$

*where* $𝐙\sim {𝒩}_{p}(0,\Sigma^{-1}(\beta ))$ *and* k=q−2.

The proof of Theorem 2 is provided in the Appendix.

Based on the Theorem 2 we can easily obtain the ADB, ADQB, ADMSE and ADQR of the estimators. First, we consider the ADB and ADQB of the estimators. Clearly,


$$\begin{array}{cc} \text{(i)}\;b_{1}({\hat{\beta }}_{n}^{pl}) & =\lim_{n\to \infty }E\{\sqrt{n}({\hat{\beta }}_{n}^{pl}-\beta )\}=0\;\text{and}\;B_{1}({\hat{\beta }}_{n}^{pl})=0, \end{array}$$ (13)

$$\begin{array}{cc} \text{(ii)}\;b_{2}({\hat{\beta }}_{n}^{R}) & =\lim_{n\to \infty }E\{\sqrt{n}({\hat{\beta }}_{n}^{R}-\beta )\}=-\delta \;\text{and}\;B_{2}({\hat{\beta }}_{n}^{R})=\delta^{\prime }\Sigma (\beta )\delta =\Delta^{2},\\ \text{(iii)}\;b_{3}({\hat{\beta }}_{n}^{R}) & =\lim_{n\to \infty }E\{\sqrt{n}({\hat{\beta }}_{n}^{R}-\beta )\}=\lim_{n\to \infty }E\{-\sqrt{n}({\hat{\beta }}_{n}^{pl}-\beta_{n}^{R})I({𝒲}_{n}<{𝒲}_{n,\alpha })\}\\ & =-\delta H_{q+2}(\chi_{q}^{2}(\alpha );\Delta^{2}),\text{ since }{𝒲}_{n,\alpha }\overset{d\;}{\to }\chi_{q}^{2}(\alpha )\text{ as }n\to \infty ,\\ {𝐁}_{3}({\hat{\beta }}_{n}^{PTE}) & =\Delta^{2}\{H_{q+2}(\chi_{q}^{2}(\alpha );\Delta^{2}){\}}^{2}.\\ \text{(iv)}\;b_{4}({\hat{\beta }}_{n}^{S}) & =\lim_{n\to \infty }E\{\sqrt{n}({\hat{\beta }}_{n}^{S}-\beta )\}=-k\lim_{n\to \infty }E\{\sqrt{n}({\hat{\beta }}_{n}^{pl}-\beta_{n}^{R}){𝒲}_{n}^{-1}\}\\ & =-k\delta E[\chi_{q+2}^{-2}(\Delta^{2})],\;k=\lim_{n\to \infty }k_{n}=(q-2) \end{array}$$ (14)

and $B_{4}({\hat{\beta }}_{n}^{S})=k^{2}\Delta^{2}\{E[\chi_{q+2}^{-2}(\Delta^{2})]{\}}^{2}.$ Similarly,


$$\begin{array}{cc} \text{(v)}\;b_{5}({\hat{\beta }}_{n}^{S+}) & =\lim_{n\to \infty }E\{\sqrt{n}({\hat{\beta }}_{n}^{S+}-\beta )\}\\ & =\lim_{n\to \infty }E\{\sqrt{n}({\hat{\beta }}_{n}^{S}-\beta )-\sqrt{n}({\hat{\beta }}_{n}^{pl}-\beta_{n}^{R})(1-k_{n}{𝒲}_{n}^{-1})I({𝒲}_{n}<k_{n})\}\\ & =b_{4}({\hat{\beta }}_{n}^{S})-\delta E\{(1-k\chi_{q+2}^{-2}(\Delta^{2}))I(\chi_{q+2}^{2}(\Delta^{2})<k)\},\\ B_{5}({\hat{\beta }}_{n}^{S+}) & =\Delta^{2}\{kE[\chi_{q+2}^{-2}(\Delta^{2})]-E[(1-k\chi_{q+2}^{-2}(\Delta^{2}){)}^{2}I(\chi_{q+2}^{2}(\Delta^{2})<k)]{\}}^{2}. \end{array}$$ (15)

We derive the expressions for the ADQR and ADMSE of the estimators, as well as the ADMSE, based on Theorem 2. The complete proofs are provided in the Appendix.

**Remark 1.** *The asymptotic structure derived in Theorem 2 is based on martingale central limit theorems for counting processes (see, e.g., Andersen and Gill, 1982). The present results extend these classical asymptotic arguments in several directions that are particularly relevant in the context of the time-dependent Cox model.*

*First, the analysis is carried out within a time-dependent Cox regression framework, where the covariates are stochastic processes. This setting introduces additional technical challenges due to the presence of counting processes and stochastic integrals.*

*Second, Theorem 2 establishes the joint asymptotic distribution of the unrestricted and restricted estimators, as well as their differences. This joint characterization plays a key role in deriving the asymptotic properties of the proposed shrinkage estimators and is not commonly developed in standard treatments.*

*Third, the result provides explicit asymptotic representations under local alternatives, which enable a unified derivation of ADB, ADQB, ADMSE, and ADQR for all estimators under consideration.*

*A comprehensive framework that simultaneously incorporates time-dependent covariates, linear restrictions, and Stein-type shrinkage estimation does not appear to have been systematically studied in the existing literature.*

## Simulation study

We conduct a Monte Carlo simulation study to assess the finite-sample behavior of the proposed shrinkage estimators in the time-dependent Cox model. The comparison includes the UE, RE, PTE, SE, and PSE, together with three widely used penalized likelihood estimators—LASSO [14], ALASSO [21], and ENET [22]—thereby enabling a comprehensive evaluation of classical, shrinkage-based, and regularized approaches.

Two practical considerations guide the simulation design. First, time-dependent covariates may induce immortal-time bias if risk intervals are not constructed correctly; to avoid this, all datasets are generated directly in the (start,stop,status) counting-process format [23]. Second, extremely large coefficients can lead to quasi-separation and numerical instability in the partial likelihood [19]; thus, coefficient magnitudes are restricted to moderate values. These adjustments ensure realistic hazard trajectories and unbiased assessment of estimator performance.

We consider the time-dependent Cox model (1) with parameter vector $\beta =(\beta_{1}^{\prime },\beta_{2}^{\prime }{)}^{\prime }$, where $\beta_{1}=(1.1,-1.2,1.4,0.9{)}^{\prime }$ and $\beta_{2}=0_{p_{2}}^{\prime }$. Covariates follow $X_{i}(t)\sim N_{p}(0,0.1I_{p})$ with constant baseline hazard *h*_0_(*t*)=0.1. Survival times are generated via the inverse transform method,


$$\begin{array}{c} T_{i}=-\frac{\log U_{i}}{h_{0}(t)\exp(\beta^{\prime }X_{i}(t))},\;U_{i}\sim (0,1), \end{array}$$

and censoring times are drawn to yield approximately 15% censoring. Each trajectory is partitioned into three equal-length intervals to create a valid counting-process representation. Departures from the null constraint $H_{0}:H\beta =0$ are introduced using the deviation index $\Delta^{*}=\|\beta -\beta_{0}\|,$ where $\beta_{0}=((1.1,-1.2,1.4,0.9{)}^{\prime },0_{p_{2}}^{\prime }{)}^{\prime }$ and ‖·‖ denotes the Euclidean norm. For example, when $\Delta^{*}=0.5$, the coefficient vector takes the form $\beta =((1.1,-1.2,1.4,0.9{)}^{\prime },0.5,0_{p_{2}-1}^{\prime }{)}^{\prime }$..

Simulation scenarios vary over sample sizes n∈{200,300,500} and numbers of potentially irrelevant covariates $p_{2}\in \{4,11\}$, with 100 replications per configuration. A key methodological component is the adaptive selection of the Stein shrinkage parameter $d_{opt}$. This parameter is chosen through a short pilot Monte Carlo procedure that estimates the empirical distribution of the Wald statistic and constructs a data-driven grid of candidate values. The value minimizing the Monte Carlo MSE of the positive-rule Stein estimator is selected (Algorithm 1).

**Algorithm 1: Monte Carlo–Based Selection of $d_{opt}$**



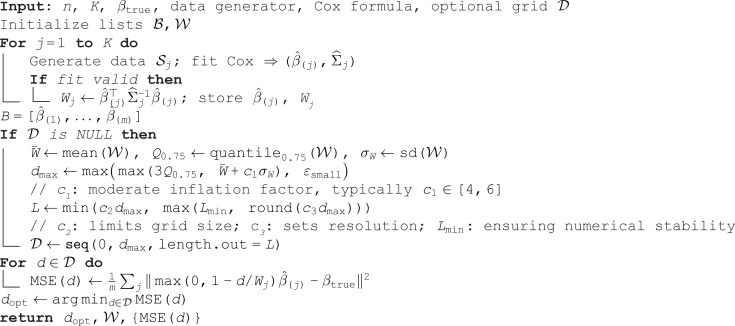



The performance of the estimators is evaluated using standard finite-sample criteria, with the mean squared error (MSE) and the simulated relative efficiency (SREF) computed as follows:


$$\begin{array}{c} MSE=\frac{1}{K}\sum_{k=1}^{K}\|{\hat{\beta }}^{\left(k\right)}-\beta {\|}^{2},\;SREF=\frac{MSE({\hat{\beta }}_{UE})}{MSE(\hat{\beta })}. \end{array}$$

Coefficientwise empirical bias and standard deviation are also reported to assess systematic deviations and sampling variability. Together, these measures provide a coherent evaluation of the precision, robustness, and efficiency of the competing estimators across a broad range of data-generating mechanisms.

**Fig 1:**
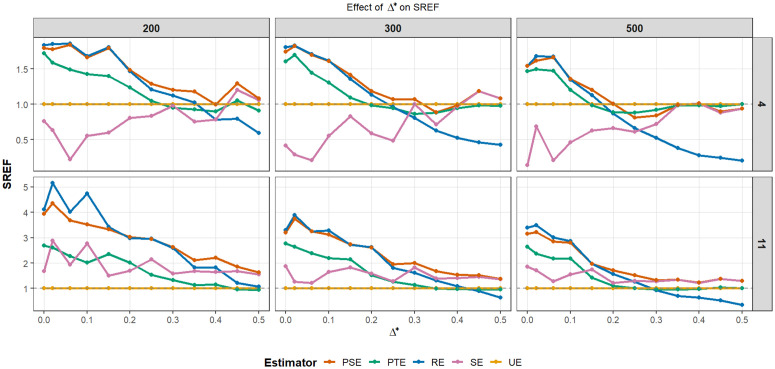
Efficiency of UE, RE, PTE, SE, and PSE estimators versus $\Delta^{*}$. Results are shown across different sample sizes and values of *p*_2_. Efficiency is calculated relative to the UE estimator.

**Fig 2:**
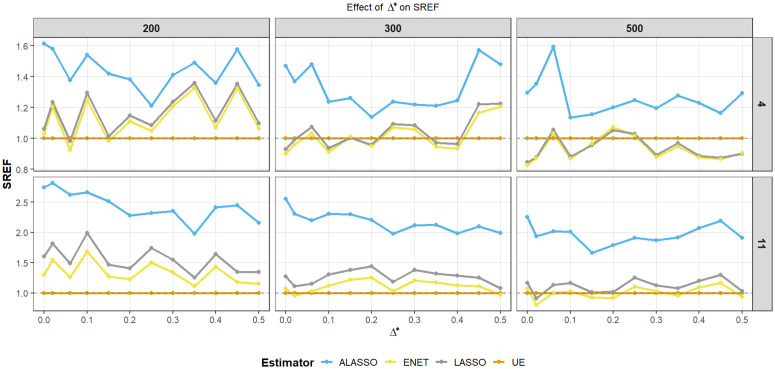
Efficiency of UE, LASSO, ALASSO, and ENET estimators versus $\Delta^{*}$. Results are presented across different sample sizes and values of *p*_2_. Relative efficiency is computed with respect to the UE estimator.

**Fig 3:**
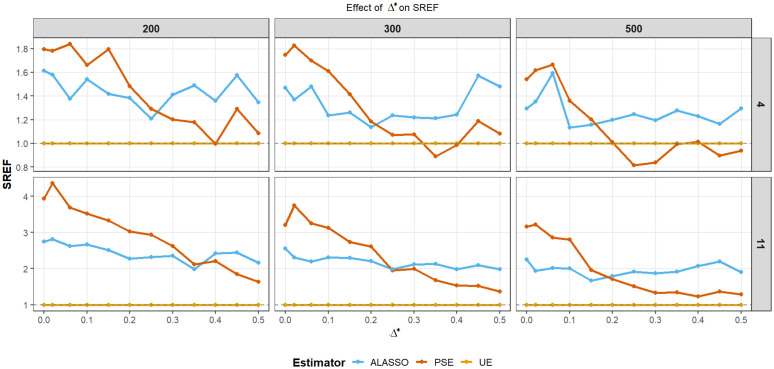
Efficiency of UE, ALASSO, and PSE estimators versus $\Delta^{*}$. Results are displayed across different sample sizes and values of *p*_2_. Relative efficiency is calculated with respect to the UE estimator.

Table 1 and Figs 1–3 provide a unified assessment of the finite-sample performance of all estimators and empirically validate the theoretical results presented in *Shrinkage Estimation under the Time-Dependent Cox Model*. The UE exhibits small biases and standard deviations across all sample sizes and dimensions (Table 1), confirming both the numerical stability of the simulation design and the suitability of its empirical SDs as a reference scale for interpreting $\Delta^{*}$. Notably, the largest deviations considered ($\Delta^{*}\approx 0.4-0.5$) correspond to nearly ten times the empirical SD of ${\hat{\beta }}_{5}$.

**Table 1:** Estimated biases and standard deviations (SDs) of regression coefficients $\beta_{1}--\beta_{5}$. Results are shown for different sample sizes and values of *p*_2_.

n	*p*_2_	$\beta_{1}$		$\beta_{2}$		$\beta_{3}$		$\beta_{4}$		$\beta_{5}$	
		Bias	SD	Bias	SD	Bias	SD	Bias	SD	Bias	SD
200	4	0.044	0.020	−0.067	0.019	0.062	0.019	0.032	0.017	−0.008	0.013
300	4	0.036	0.013	−0.036	0.015	0.027	0.015	0.010	0.013	0.010	0.010
500	4	0.006	0.010	−0.011	0.011	0.010	0.011	0.012	0.009	−0.006	0.007
200	11	0.115	0.019	−0.109	0.020	0.153	0.020	0.088	0.017	−0.004	0.014
300	11	0.061	0.013	−0.092	0.013	0.079	0.016	0.053	0.014	0.002	0.011
500	11	0.049	0.011	−0.051	0.011	0.059	0.011	0.030	0.009	0.001	0.008

Bias: empirical bias of the estimator; SD: empirical standard deviation.

Fig 1 shows that the PSE uniformly dominates the UE and PTE and performs on par with or better than the SE across most deviations. The RE attains the highest efficiency only when $\Delta^{*}$ is close to zero, reflecting the near-validity of the restriction Hβ=0; however, its efficiency deteriorates rapidly once the restriction is violated. In contrast, the PSE remains robust across a broad range of departures, fully consistent with its optimal ADMSE and ADQR properties.

Fig 2 compares the UE with penalized estimators. ALASSO achieves the highest efficiency near $\Delta^{*}=0$, owing to its adaptive weighting strategy, but all penalized estimators lose efficiency as $\Delta^{*}$ increases. When considered alongside Fig 1, ALASSO surpasses the PSE only under sufficiently large deviations where the restriction is clearly invalid.

Fig 3 further contrasts PSE and ALASSO across various (*n*,*p*_2_) configurations. For small $\Delta^{*}$, PSE consistently outperforms ALASSO and UE, and this dominance persists until the deviation reaches roughly an order of magnitude larger than the empirical SD of ${\hat{\beta }}_{5}$. Beyond this point, ALASSO begins to dominate, particularly in high-dimensional and small-sample settings (e.g., *p* = 15, *n* = 200), where penalization compensates for the invalidity of the restriction.

Across all scenarios, a consistent pattern emerges: the relative efficiency of both PSE and ALASSO improves as dimensionality increases or sample size decreases, reflecting the stronger stabilizing influence of shrinkage and penalization when information is limited. Additional experiments (not shown) confirm that the same trend holds for *n* < 200 and *n* > 500. For very small samples (*n* < 100), the inflated sampling variability shifts the PSE–ALASSO crossover point toward larger $\Delta^{*}$ values (often near 0.5), supporting the empirical rule that ALASSO typically overtakes PSE only when the deviation exceeds approximately ten times the SD of the relevant coefficient estimates.

Overall, these findings demonstrate that PSE achieves the most favorable bias–variance trade-off across a broad range of practically relevant settings, occupying an effective middle ground between restricted and penalized estimators. The resulting efficiency hierarchy may be summarized as $\text{ For small }\Delta^{*}:\text{RE}>\text{PSE}\approx \text{SE}>\text{PTE}>\text{UE},\text{ For large }\Delta^{*}:\text{ALASSO}>\text{PSE}>\text{UE}>\text{RE}$.

**Fig 4:**
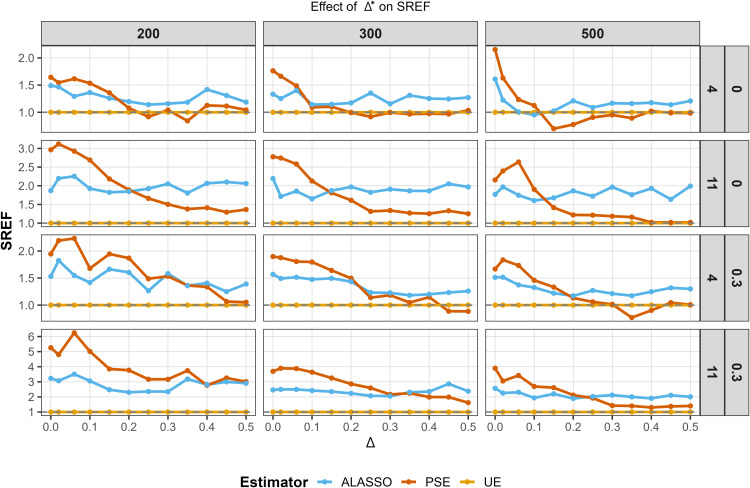
Efficiency of UE, ALASSO, and PSE estimators versus $\Delta^{*}$. Results are shown for different sample sizes, values of *p*_2_, and censoring rates. Relative efficiency is computed with respect to the UE estimator.

**Additional Censoring-Level Experiments (Fig 4).** To further examine robustness to censoring, an additional experiment was conducted under two scenarios: (i) no censoring and (ii) a higher censoring rate of 30%. Fig 4 compares the efficiencies of PSE, ALASSO, and UE in both settings. The overall qualitative patterns mirror those observed under the 15% censoring design: PSE dominates for small and moderate deviations, while ALASSO overtakes PSE only for sufficiently large $\Delta^{*}$.

A notable additional finding is that higher censoring amplifies the relative advantage of PSE at larger deviations. Specifically, as the censoring rate increases, the efficiency crossover point shifts to the right, indicating that PSE retains superior performance over a broader range of departures from *H*_0_. This behavior highlights the resilience of PSE in scenarios where information loss due to censoring is more pronounced.

## Application to the Mayo Clinic PBC data

We illustrate the proposed estimators using the Mayo Clinic PBC dataset [24], derived from a randomized clinical trial of D-penicillamine. The analysis includes 312 patients with complete longitudinal records, reformatted into $\left(t_{\text{start}},t_{\text{stop}}\right]$ intervals using tmerge(), resulting in a censoring rate of approximately 53.8%. The dataset contains thirteen demographic and biochemical predictors measured repeatedly over time; a concise description is provided in Table 2.

**Table 2:** List of variables in the pbcseq dataset.

Variable	Description
*id*	Patient identifier (1–312)
*day*	Days since enrollment at each follow-up visit
*time*	Days to death, transplantation, or censoring
*status*	Event indicator (0 = censored, 1 = transplant, 2 = death)
*x*_1_: *bili*	Serum bilirubin (mg/dl)
*x*_2_: *albumin*	Serum albumin (g/dl)
*x*_3_: *protime*	Standardized prothrombin time
*x*_4_: *alk*.*phos*	Alkaline phosphatase (U/liter)
*x*_5_: *ast*	Aspartate aminotransferase (U/ml)
*x*_6_: *chol*	Serum cholesterol (mg/dl)
*x*_7_: *platelet*	Platelet count (per mm^3^/1000)
*x*_8_: *ascites*	Presence of ascites
*x*_9_: *edema*	Edema score (0, 0.5, 1)
*x*_10_: *hepato*	Hepatomegaly indicator
*x*_11_: *spiders*	Spider angiomas indicator
*x*_12_: *stage*	Histologic stage (1–4)
*x*_13_: *sex*	Gender (0 = male, 1 = female)

A preliminary BIC-based stepwise selection identified seven influential covariates—log(bili), albumin, log(protime), log(ast), hepato, stage, and sex. The remaining predictors were treated as irrelevant, defining a restricted subspace with *p*_2_ = 6. To evaluate finite-sample properties, we employed subject-level case-resampling with *B* = 500 bootstrap replicates, preserving the within-subject dependence inherent to longitudinal measurements.

Table 3 reports bootstrap estimates (Est), standard errors (SE), and simulated relative efficiencies (SREF). The Positive-rule Stein Estimator (PSE) achieved the highest overall efficiency (SREF = 1.72), followed by RE and ENET, whereas UE served as the baseline. SE and PTE showed reduced stability under moderate departures from the restriction, while PSE delivered consistently smaller SEs across several clinically relevant coefficients

**Table 3:** Bootstrap estimates and simulated relative efficiencies for significant time-dependent covariates.

Estimator	$\beta_{1}$		$\beta_{2}$		$\beta_{3}$		$\beta_{5}$		$\beta_{10}$		$\beta_{12}$		$\beta_{13}$		SREF
	Est	SE	Est	SE	Est	SE	Est	SE	Est	SE	Est	SE	Est	SE	
UE	1.397	0.020	2.082	0.083	−0.679	0.036	−1.809	0.027	0.344	0.015	−0.524	0.028	−0.590	0.032	1.000
RE	1.431	0.019	2.718	0.068	−0.755	0.032	−1.862	0.025	0.424	0.013	−0.456	0.028	−0.439	0.028	1.718
PTE	1.411	0.020	2.239	0.086	−0.700	0.036	−1.824	0.027	0.366	0.015	−0.506	0.028	−0.553	0.032	0.928
SE	1.514	0.025	3.780	0.103	−0.895	0.034	−1.960	0.035	0.565	0.020	−0.343	0.035	−0.181	0.041	0.441
PSE	1.431	0.019	2.718	0.068	−0.755	0.032	−1.862	0.025	0.424	0.013	−0.456	0.028	−0.439	0.028	1.718
LASSO	1.312	0.021	0.655	0.067	−0.377	0.036	−1.596	0.031	0.278	0.014	−0.224	0.026	0.000	0.000	1.487
ALASSO	1.307	0.018	1.340	0.080	−0.512	0.025	−1.757	0.025	0.192	0.016	−0.204	0.026	0.000	0.000	1.227
ENET	1.307	0.020	0.669	0.064	−0.380	0.033	−1.578	0.030	0.285	0.014	−0.229	0.026	0.000	0.000	1.572

Est: bootstrap mean estimate; SE: bootstrap standard error; SREF: simulated relative efficiency.

Among penalized estimators, ALASSO produced the smallest SEs overall, with ENET exhibiting efficiency comparable to PSE. LASSO demonstrated stronger shrinkage, slightly attenuating both weak and strong effects; the adaptive weighting in ALASSO mitigated this bias.

The estimated effects aligned with established clinical understanding: elevated bilirubin, prolonged prothrombin time, and advanced histologic stage were associated with increased risk, whereas higher albumin, hepatomegaly, and female sex reduced the hazard.

**Fig 5:**
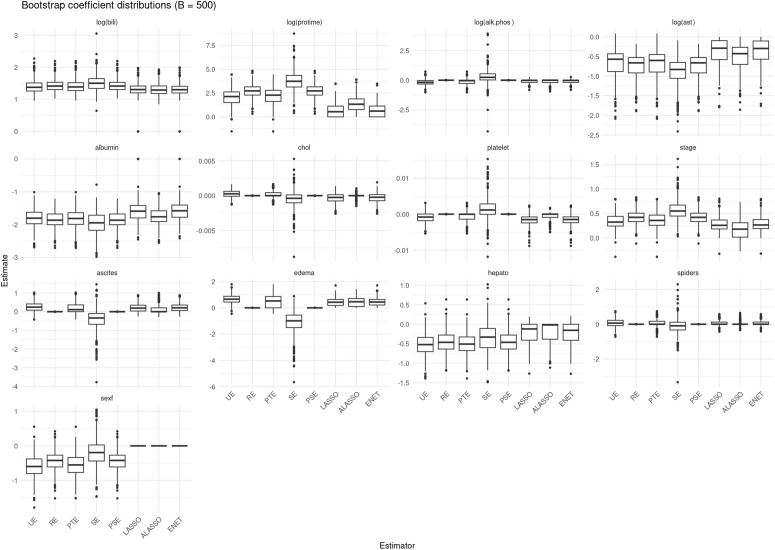
Bootstrap distributions of regression coefficient estimates in the time-dependent PBC Cox model. Boxplots are based on *B* = 500 bootstrap samples and compare eight estimation methods.

Fig 5 depicts the bootstrap distributions of the coefficient estimates. PSE and ENET exhibit the narrowest dispersion, ALASSO shows comparably stable behavior, while LASSO displays overshrinkage and heavier tails for weaker predictors. UE yields the greatest variability. These graphical results reinforce the superior finite-sample efficiency and robustness of adaptive and positive-rule shrinkage estimators in time-dependent Cox regression with partial prior information.

## Conclusions

This work develops a unified class of improved estimators for the time-dependent Cox proportional hazards model by incorporating linear subspace information through preliminary-test and Stein-type shrinkage strategies. Within the counting-process framework, we established the asymptotic distributions of the proposed estimators and derived explicit expressions for their asymptotic bias and risk under local alternatives, thereby elucidating the conditions under which principled shrinkage yields efficiency gains.

Monte Carlo simulations and the longitudinal Mayo Clinic PBC analysis provide strong empirical validation of the theoretical results. Across a wide range of settings, the positive-rule Stein estimator (PSE) achieves the most favorable bias–variance trade-off whenever the imposed restriction is approximately correct, and maintains competitive performance under moderate misspecification. A recurring empirical finding is that adaptive LASSO surpasses PSE only when the deviation from the restriction exceeds roughly an order of magnitude of the empirical standard deviation of the associated coefficient, offering a practical rule-of-thumb for applied analyses.

In summary, positive-rule Stein shrinkage constitutes a robust and interpretable alternative to both unrestricted and penalized estimators in semiparametric survival models with time-dependent covariates. The proposed framework suggests several directions for future research, including hybrid shrinkage–penalization approaches, data-adaptive selection of shrinkage intensity, and extensions to high-dimensional or partially sparse Cox-type models.

## Appendix

### A Proofs of main results

#### A.1 Proof of Theorem 1.

Let **a** be any vector and **B** be any positive definite matrix. Define the quadratic form


$$\begin{array}{c} \|𝐚{\|}_{𝐁}^{2}={𝐚}^{⊤}𝐁𝐚. \end{array}$$

Moreover, let ${𝒲}_{n,\alpha }$ denote the upper α-quantile of the asymptotic $\chi_{q}^{2}$ distribution of the Wald statistic ${𝒲}_{n}$. Finally, let *k*_*n*_ be the sequence of shrinkage constants appearing in the definition of the Stein-type estimator, satisfying $k_{n}\to k=q-2$ as n→∞.

We first consider the quadratic form of the difference between ${\hat{\beta }}_{n}^{PTE}$ and ${\hat{\beta }}_{n}^{pl}$,


$$\begin{array}{c} {\left\|{\hat{\beta }}_{n}^{PTE}-{\hat{\beta }}_{n}^{pl}\right\|}_{I_{n}({\hat{\beta }}_{n}^{pl})}^{2}={𝒲}_{n}I({𝒲}_{n}<{𝒲}_{n,\alpha })\leq {𝒲}_{n,\alpha }I({𝒲}_{n}<{𝒲}_{n,\alpha }). \end{array}$$

Under the fixed alternative $K_{\pi }$, we have ${𝒲}_{n}\overset{P\;}{\to }\infty$, which implies


$$\begin{array}{c} P({𝒲}_{n}<{𝒲}_{n,\alpha })\to 0. \end{array}$$

Since ${𝒲}_{n,\alpha }$ is a fixed constant, we obtain


$$\begin{array}{c} 𝔼\{{𝒲}_{n,\alpha }I({𝒲}_{n}<{𝒲}_{n,\alpha })\}\leq {𝒲}_{n,\alpha }P({𝒲}_{n}<{𝒲}_{n,\alpha })\to 0. \end{array}$$

Therefore,


$$\begin{array}{c} \lim_{n\to \infty }𝔼\left\{{𝒲}_{n,\alpha }I({𝒲}_{n}<{𝒲}_{n,\alpha })\right\}=0, \end{array}$$

which proves part (i). Next, we consider the Stein-type estimator. We have


$$\begin{array}{c} {\left\|{\hat{\beta }}_{n}^{S}-{\hat{\beta }}_{n}^{pl}\right\|}_{I_{n}({\hat{\beta }}_{n}^{pl})}^{2}=k_{n}^{2}{\left\|{\hat{\beta }}_{n}^{R}-{\hat{\beta }}_{n}^{pl}\right\|}_{I_{n}({\hat{\beta }}_{n}^{pl})}^{2}{𝒲}_{n}^{-2}=k_{n}^{2}{𝒲}_{n}^{-1}. \end{array}$$

Under the fixed alternative $K_{\pi }$, we have ${𝒲}_{n}\overset{P\;}{\to }\infty$, which implies that ${𝒲}_{n}^{-1}\overset{P\;}{\to }0$. Moreover, since ${𝒲}_{n}^{-1}\leq 1$ for sufficiently large *n*, it follows by the dominated convergence theorem that


$$\begin{array}{c} 𝔼({𝒲}_{n}^{-1})\to 0. \end{array}$$

Since $k_{n}\to k$, we obtain


$$\begin{array}{c} 𝔼\left\{{\left\|{\hat{\beta }}_{n}^{S}-{\hat{\beta }}_{n}^{pl}\right\|}_{I_{n}({\hat{\beta }}_{n}^{pl})}^{2}\right\}\to 0. \end{array}$$

Consequently,


$$\begin{array}{c} \sqrt{n}I_{n}^{1/2}({\hat{\beta }}_{n}^{pl})({\hat{\beta }}_{n}^{S}-\beta )=\sqrt{n}I_{n}^{1/2}(\beta )({\hat{\beta }}_{n}^{R}-\beta )+o_{P}(1). \end{array}$$

Finally, for the positive-part Stein estimator, we have


$$\begin{array}{cc} {\left\|{\hat{\beta }}_{n}^{S+}-{\hat{\beta }}_{n}^{pl}\right\|}_{I_{n}({\hat{\beta }}_{n}^{pl})}^{2} & =k_{n}^{2}{𝒲}_{n}^{-1}+{𝒲}_{n}(1-k_{n}{𝒲}_{n}^{-1}{)}^{2}I({𝒲}_{n}<k_{n})\\ & \;-2k_{n}^{2}{𝒲}_{n}(1-k_{n}{𝒲}_{n}^{-1})I({𝒲}_{n}<k_{n}). \end{array}$$

Each term on the right-hand side can be treated similarly to the previous cases. In particular, using the fact that ${𝒲}_{n}\overset{P\;}{\to }\infty$ and $k_{n}\to k$, together with boundedness arguments, we obtain


$$\begin{array}{c} 𝔼\left\{{\left\|{\hat{\beta }}_{n}^{S+}-{\hat{\beta }}_{n}^{pl}\right\|}_{I_{n}({\hat{\beta }}_{n}^{pl})}^{2}\right\}\to 0. \end{array}$$

Hence,


$$\begin{array}{c} \sqrt{n}I_{n}^{1/2}({\hat{\beta }}_{n}^{pl})({\hat{\beta }}_{n}^{S+}-\beta )=\sqrt{n}I_{n}^{1/2}(\beta )({\hat{\beta }}_{n}^{pl}-\beta )+o_{P}(1).\;\text{Q.E.D.} \end{array}$$

#### A.2 Proof Equation 11.

Let’s compute the expression $\sqrt{n}I_{n}^{1/2}({\hat{\beta }}_{n}^{pl})({\hat{\beta }}_{n}^{R}-\beta )$ using the given equation for ${\hat{\beta }}_{n}^{R}$ and considering the alternative hypothesis $K_{\pi }:H\beta =h+\pi$.

The equation for the restricted estimator is:


$$\begin{array}{c} {\hat{\beta }}_{n}^{R}={\hat{\beta }}_{n}^{pl}-I^{-1}({\hat{\beta }}_{n}^{pl})H^{\prime }(HI^{-1}({\hat{\beta }}_{n}^{pl})H^{\prime }{)}^{-1}(H{\hat{\beta }}_{n}^{pl}-h) \end{array}$$

First, let’s find the difference $\left({\hat{\beta }}_{n}^{R}-\beta \right)$:


$$\begin{array}{c} {\hat{\beta }}_{n}^{R}-\beta ={\hat{\beta }}_{n}^{pl}-\beta -I^{-1}({\hat{\beta }}_{n}^{pl})H^{\prime }(HI^{-1}({\hat{\beta }}_{n}^{pl})H^{\prime }{)}^{-1}(H{\hat{\beta }}_{n}^{pl}-h) \end{array}$$

Now, we need to substitute $H{\hat{\beta }}_{n}^{pl}-h$. We can write this as:


$$\begin{array}{c} H{\hat{\beta }}_{n}^{pl}-h=H{\hat{\beta }}_{n}^{pl}-H\beta +H\beta -h=H({\hat{\beta }}_{n}^{pl}-\beta )+(H\beta -h) \end{array}$$

Under the alternative hypothesis $K_{\pi }:𝐇\beta =h+\pi$, it follows that $𝐇{\hat{\beta }}_{n}^{pl}-h=𝐇({\hat{\beta }}_{n}^{pl}-\beta )+\pi$. Now, substitute this back into the expression for $\left({\hat{\beta }}_{n}^{R}-\beta \right)$:


$$\begin{array}{c} {\hat{\beta }}_{n}^{R}-\beta ={\hat{\beta }}_{n}^{pl}-\beta -I^{-1}({\hat{\beta }}_{n}^{pl})H^{\prime }(HI^{-1}({\hat{\beta }}_{n}^{pl})H^{\prime }{)}^{-1}(H({\hat{\beta }}_{n}^{pl}-\beta )+\pi ) \end{array}$$

Finally, we multiply this entire expression by $\sqrt{n}I_{n}^{1/2}({\hat{\beta }}_{n}^{pl})$:


$$\begin{array}{c} \sqrt{n}I_{n}^{1/2}({\hat{\beta }}_{n}^{pl})({\hat{\beta }}_{n}^{R}-\beta )=\sqrt{n}I_{n}^{1/2}({\hat{\beta }}_{n}^{pl})({\hat{\beta }}_{n}^{pl}-\beta )- \end{array}$$

$$\begin{array}{c} \sqrt{n}I_{n}^{1/2}({\hat{\beta }}_{n}^{pl})I^{-1}({\hat{\beta }}_{n}^{pl})H^{\prime }(HI^{-1}({\hat{\beta }}_{n}^{pl})H^{\prime }{)}^{-1}(H({\hat{\beta }}_{n}^{pl}-\beta )+\pi ) \end{array}$$

We can simplify the second term slightly: $I_{n}^{1/2}({\hat{\beta }}_{n}^{pl})I^{-1}({\hat{\beta }}_{n}^{pl})=I_{n}^{-1/2}({\hat{\beta }}_{n}^{pl})$. So the expression becomes:


$$\begin{array}{c} \sqrt{n}I_{n}^{1/2}({\hat{\beta }}_{n}^{pl})({\hat{\beta }}_{n}^{R}-\beta )=\sqrt{n}I_{n}^{1/2}({\hat{\beta }}_{n}^{pl})({\hat{\beta }}_{n}^{pl}-\beta )- \end{array}$$

$$\begin{array}{c} \sqrt{n}I_{n}^{1/2}({\hat{\beta }}_{n}^{pl})H^{\prime }(HI^{-1}({\hat{\beta }}_{n}^{pl})H^{\prime }{)}^{-1}(H({\hat{\beta }}_{n}^{pl}-\beta )+\pi ) \end{array}$$

This is the computed expression for $\sqrt{n}I_{n}^{1/2}({\hat{\beta }}_{n}^{pl})({\hat{\beta }}_{n}^{R}-\beta )$ in terms of ${\hat{\beta }}_{n}^{pl}$, β, ***H***, ***h***, π, and the information matrix $I_{n}({\hat{\beta }}_{n}^{pl})$.

#### A.3 Proof of Theorem 2.

The proof follows standard martingale central limit arguments (see Andersen and Gill, 1982 [2]); however, its application in the present setting requires careful handling of the time-dependent covariates, counting process structure, and the imposed linear restrictions.

(i) See Theorem 4.2 of [2].
(ii) $V_{n}^{\left(2\right)}=\sqrt{n}({\hat{\beta }}_{n}^{R}-\beta )=\sqrt{n}\left\{({\hat{\beta }}_{n}^{pl}-\beta )-I^{-1}({\hat{\beta }}_{n}^{pl})H^{⊤}(HI^{-1}({\hat{\beta }}_{n}^{pl})H^{⊤}{)}^{-1}(H{\hat{\beta }}_{n}^{pl}-h)\right\}$ $=\sqrt{n}({\hat{\beta }}_{n}^{pl}-\beta )-I^{-1}({\hat{\beta }}_{n}^{pl})H^{⊤}(HI^{-1}({\hat{\beta }}_{n}^{pl})H^{⊤}{)}^{-1}H[\sqrt{n}({\hat{\beta }}_{n}^{pl}-\beta )+\pi ]$. By (i) $V_{n}^{\left(2\right)}\sim N_{p}(-\delta ,𝐀)$ as n→∞.
(iii) $V_{n}^{\left(3\right)}=\sqrt{n}({\hat{\beta }}_{n}^{pl}-{\hat{\beta }}_{n}^{R})=I^{-1}({\hat{\beta }}_{n}^{pl})H^{⊤}(HI^{-1}({\hat{\beta }}_{n}^{pl})H^{⊤}{)}^{-1}H[\sqrt{n}({\hat{\beta }}_{n}^{R}-\beta )+\pi ]\sim N_{p}(\delta ,\Sigma (\beta )-A)$ as n→∞.
(iv) and (v) follow similarly. To prove (vi), we note that $\sqrt{n}I^{1/2}({\hat{\beta }}_{n}^{pl})(H{\hat{\beta }}_{n}^{pl}-h)\overset{d}{⋍}N_{q}(\Sigma^{1/2}(\beta )\pi ,(H\Sigma^{-1}(\beta )H^{⊤}))$ as n→∞. Hence,
  $$\begin{array}{c} n(H{\hat{\beta }}_{n}^{pl}-h{)}^{⊤}(HI^{-1}({\hat{\beta }}_{n}^{pl})H^{⊤}{)}^{-1}(H{\hat{\beta }}_{n}^{pl}-h)\overset{d}{=}\chi_{q}^{2}(\Delta^{2}) \end{array}$$
  as n→∞ where $\Delta^{2}=\pi^{⊤}(H\Sigma^{-1}(\beta )H^{⊤}{)}^{-1}\pi =\delta^{⊤}\Sigma (\beta )\delta$.
(vi) $\lim_{n\to \infty }P\left\{\sqrt{n}({\hat{\beta }}_{n}^{PTE}-\beta )\leq x\right\}=\lim_{n\to \infty }P\left\{\sqrt{n}({\hat{\beta }}_{n}^{R}-\beta )\leq x,{𝒲}_{n}\leq {𝒲}_{n,\alpha }\right\}$
  $+\lim_{n\to \infty }P\left\{\sqrt{n}({\hat{\beta }}_{n}^{pl}-\beta )\leq x;{𝒲}_{n}>{𝒲}_{n},\alpha \right\}$.

Since $\sqrt{n}({\hat{\beta }}_{n}^{R}-\beta )$ and $\sqrt{n}({\hat{\beta }}_{n}^{pl}-\beta )$ are independent, the first term reduces to $H_{q}(\chi_{q}^{2}(\alpha );\Delta^{2})\Phi_{p}(x+\delta ;0,A)$ as n→∞. The second term is obtained by conditional arguments as n→∞ and is given by


$$\begin{array}{c} \int_{E(\delta )}\Phi_{p}(x-\Sigma^{-1}(\beta )H^{⊤}(H\Sigma (\beta )H^{⊤}{)}^{-1}Z;0,[\Sigma (\beta )-A]) \end{array}$$

$$\begin{array}{c} \times d\Phi_{p}(Z;0,(H\Sigma (\beta )H^{⊤})), \end{array}$$

where $E(\delta )=\{Z;(Z+\delta {)}^{⊤}(H\Sigma (\beta )H^{⊤}{)}^{-1}(Z+\delta )\geq \chi_{q}^{2}(\alpha )\}$.

Proofs of (viii) and (ix) are obtained by writing the expressions in terms of $(H\beta -h)=n^{-1/2}\pi$ and then applying the distribution of the related statistics.

#### A.4 Proofs for $R_{3}({\hat{\beta }}_{n}^{PTE};W),R_{4}({\hat{\beta }}_{n}^{S};W)$ and $R_{5}({\hat{\beta }}_{n}^{S+};W)$ equations.


$$\begin{array}{cc} R_{3}({\hat{\beta }}_{n}^{PTE};W) & =\lim_{n\to \infty }E[n({\hat{\beta }}_{n}^{PTE}-\beta {)}^{⊤}W({\hat{\beta }}_{n}^{PTE}-\beta )]\\ & =\lim_{n\to \infty }E[n({\hat{\beta }}_{n}^{pl}-\beta {)}^{⊤}W({\hat{\beta }}_{n}^{pl}-\beta )]\\ & \;+\lim_{n\to \infty }E[n({\hat{\beta }}_{n}^{pl}-{\hat{\beta }}_{n}^{R}{)}^{⊤}W({\hat{\beta }}_{n}^{pl}-{\hat{\beta }}_{n}^{R})I({𝒲}_{n}<{𝒲}_{n,\alpha })]\\ & \;-2\lim_{n\to \infty }E[n({\hat{\beta }}_{n}^{pl}-\beta {)}^{⊤}W({\hat{\beta }}_{n}^{pl}-{\hat{\beta }}_{n}^{R})I({𝒲}_{n}<{𝒲}_{n,\alpha })]\\ & =R_{1}({\hat{\beta }}_{n}^{pl};W)-\lim_{n\to \infty }E[n({\hat{\beta }}_{n}^{pl}-{\hat{\beta }}_{n}^{R}{)}^{⊤}W({\hat{\beta }}_{n}^{pl}-{\hat{\beta }}_{n}^{R})I({𝒲}_{n}<{𝒲}_{n,\alpha })]\\ & \;+2\eta^{⊤}W\{\lim_{n\to \infty }E[\sqrt{n}({\hat{\beta }}_{n}^{pl}-{\hat{\beta }}_{n}^{R})I({𝒲}_{n}>{𝒲}_{n,\alpha })]\}\\ & =R_{1}({\hat{\beta }}_{n}^{pl};W)-\text{tr}(\Sigma (\beta {)}^{-1}H^{⊤}(H\Sigma (\beta {)}^{-1}H^{⊤}{)}^{-1}H\Sigma (\beta {)}^{-1}W)\\ & \times H_{q+2}(\chi_{q}^{2}(\alpha );\Delta^{2})+(\delta^{⊤}W\delta )\{2H_{q+2}(\chi_{q}^{2}(\alpha );\Delta^{2})\\ & -H_{q+4}(\chi_{q}^{2}(\alpha );\Delta^{2})\}. \end{array}$$

Since $\Sigma (\beta {)}^{-1/2}H^{⊤}(H\Sigma (\beta {)}^{-1}H^{⊤}{)}^{-1}H\Sigma (\beta {)}^{-1/2}$ is a symmetric idempotent matrix with rank q(≤p), there exists an orthogonal matrix Γ such that


$$\begin{array}{c} \Gamma \Sigma (\beta {)}^{-1/2}H^{⊤}(H\Sigma (\beta {)}^{-1}H^{⊤}{)}^{-1}H\Sigma (\beta {)}^{-1/2}\Gamma^{⊤}=(\begin{array}{cc} {𝐈}_{q} & 0\\ 0 & 0 \end{array}), \end{array}$$

$$\begin{array}{c} \Gamma \Sigma (\beta {)}^{-1/2}W\Sigma (\beta {)}^{-1/2}\Gamma^{⊤}=(\begin{array}{cc} {𝐀}_{11} & {𝐀}_{12}\\ {𝐀}_{12}^{⊤} & {𝐀}_{22} \end{array}). \end{array}$$

The matrices **A**_11_ and **A**_12_ are of order *q* and p−q, respectively. Hence,


$$\begin{array}{cc} & \text{tr}[\Sigma (\beta {)}^{-1}H^{⊤}(H\Sigma (\beta {)}^{-1}H^{⊤}{)}^{-1}H\Sigma (\beta {)}^{-1}W]\\ & =\text{tr}[\Gamma \Sigma (\beta {)}^{-1/2}W\Sigma (\beta {)}^{-1/2}\Gamma^{⊤}(\Gamma \Sigma (\beta {)}^{-1/2}H^{⊤}(H\Sigma (\beta {)}^{-1}H^{⊤}{)}^{-1}H\Sigma (\beta {)}^{-1/2}\Gamma^{⊤})]\\ & =\text{tr}\left[(\begin{array}{cc} {𝐀}_{11} & {𝐀}_{12}\\ {𝐀}_{12}^{⊤} & {𝐀}_{22} \end{array})(\begin{array}{cc} {𝐈}_{q} & 0\\ 0 & 0 \end{array})\right]\\ & =\text{tr}({𝐀}_{11}). \end{array}$$

Further, by Theorem 2(ii),


$$\begin{array}{cc} \delta^{⊤}W\delta & =\pi^{⊤}(H\Sigma (\beta {)}^{-1}H^{⊤}{)}^{-1}H\Sigma (\beta {)}^{-1}W\Sigma (\beta {)}^{-1}H^{⊤}(H\Sigma (\beta {)}^{-1}H^{⊤}{)}^{-1}\pi \\ & =\pi^{⊤}(H\Sigma (\beta {)}^{-1}H^{⊤}{)}^{-1}H\Sigma (\beta {)}^{-1/2}\Gamma^{⊤}\Gamma \Sigma (\beta {)}^{-1/2}W\Sigma (\beta {)}^{-1/2}\Gamma^{⊤}\Gamma \\ & \times \Sigma (\beta {)}^{-1/2}H^{⊤}(H\Sigma (\beta {)}^{-1}H^{⊤}{)}^{-1}\pi \\ & =\pi^{⊤}(H\Sigma (\beta {)}^{-1}H^{⊤}{)}^{-1}H\Sigma (\beta {)}^{-1/2}\Gamma^{⊤}(\begin{array}{cc} {𝐈}_{q} & 0\\ 0 & 0 \end{array})(\begin{array}{cc} {𝐀}_{11} & {𝐀}_{12}\\ {𝐀}_{12}^{⊤} & {𝐀}_{22} \end{array})(\begin{array}{cc} {𝐈}_{q} & 0\\ 0 & 0 \end{array})\\ & \times \Gamma \Sigma (\beta {)}^{-1/2}H^{⊤}(H\Sigma (\beta {)}^{-1}H^{⊤}{)}^{-1}\pi . \end{array}$$ (16)

Let $\eta =\Gamma \Sigma (\beta {)}^{-1/2}H^{⊤}(H\Sigma (\beta {)}^{-1}H^{⊤}{)}^{-1}\pi$; then, the r.h.s. of (16) becomes


$$\begin{array}{c} \eta^{⊤}(\begin{array}{cc} {𝐀}_{11} & 0\\ 0 & 0 \end{array})\eta =\eta_{1}^{⊤}{𝐀}_{11}\eta_{1}\;\text{where}\;\eta =(\begin{array}{c} \eta_{1}\\ \eta_{2} \end{array}). \end{array}$$ (17)

Thus, $R_{3}({\hat{\beta }}_{n}^{PT};W)$ reduces to


$$\begin{array}{cc} R_{3}({\hat{\beta }}_{n}^{PT};W) & =R_{1}({\hat{\beta }}_{n}^{pl};W)-\text{tr}({𝐀}_{11})H_{q+2}(\chi_{q}^{2}(\alpha );\Delta^{2})\\ & \;+(\eta_{1}^{⊤}{𝐀}_{11}\eta_{1})\{2H_{q+2}(\chi_{q}^{2}(\alpha );\Delta^{2})-H_{q+4}(\chi_{q}^{2}(\alpha );\Delta^{2})\}. \end{array}$$

Next, we consider the risk expression for ${\hat{\beta }}_{n}^{S}$:


$$\begin{array}{cc} R_{4}({\hat{\beta }}_{n}^{S};W) & =\lim_{n\to \infty }E[n({\hat{\beta }}_{n}^{S}-\beta {)}^{⊤}W({\hat{\beta }}_{n}^{S}-\beta )]\\ & =\lim_{n\to \infty }En({\hat{\beta }}_{n}^{pl}-\beta {)}^{⊤}W({\hat{\beta }}_{n}^{pl}-\beta )\\ & \;+k^{2}\lim_{n\to \infty }E[n({\hat{\beta }}_{n}^{pl}-{\hat{\beta }}_{n}^{R}{)}^{⊤}W({\hat{\beta }}_{n}^{pl}-{\hat{\beta }}_{n}^{R}){𝒲}_{n}^{-2}]\\ & \;-2k\lim_{n\to \infty }E[n({\hat{\beta }}_{n}^{pl}-\beta {)}^{⊤}W({\hat{\beta }}_{n}^{pl}-{\hat{\beta }}_{n}^{R}){𝒲}_{n}^{-1}]\\ & =R_{1}({\hat{\beta }}_{n}^{pl};W)+k^{2}\lim_{n\to \infty }E[n({\hat{\beta }}_{n}^{pl}-{\hat{\beta }}_{n}^{R}{)}^{⊤}W({\hat{\beta }}_{n}^{pl}-{\hat{\beta }}_{n}^{R}){𝒲}_{n}^{-2}]\\ & \;-2k\lim_{n\to \infty }E\{[n({\hat{\beta }}_{n}^{pl}-\beta {)}^{⊤}W({\hat{\beta }}_{n}^{pl}-{\hat{\beta }}_{n}^{R})\\ & \;-(H\beta -h{)}^{⊤}(H\Sigma (\beta {)}^{-1}H^{⊤}{)}^{-1}H\Sigma (\beta {)}^{-1}W({\hat{\beta }}_{n}^{pl}-{\hat{\beta }}_{n}^{R})]{𝒲}_{n}^{-1}\}\\ & =R_{1}({\hat{\beta }}_{n}^{pl};W)-2k\text{tr}[\Sigma (\beta {)}^{-1}H^{⊤}(H\Sigma (\beta {)}^{-1}H^{⊤}{)}^{-1}H\Sigma (\beta {)}^{-1}W]\\ & \;-2k\pi^{⊤}(H\Sigma (\beta {)}^{-1}H^{⊤}{)}^{-1}H\Sigma (\beta {)}^{-1}W\Sigma (\beta {)}^{-1}H^{⊤}\\ & \;\times (H\Sigma (\beta {)}^{-1}H^{⊤}{)}^{-1}\pi E[\chi_{q+4}^{-2}(\Delta^{2})]+k^{2}\pi^{⊤}(H\Sigma (\beta {)}^{-1}H^{⊤}{)}^{-1}\\ & \;\times H\Sigma (\beta {)}^{-1}W\Sigma (\beta {)}^{-1}H^{⊤}(H\Sigma (\beta {)}^{-1}H^{⊤}{)}^{-1}\pi E[\chi_{q+4}^{-2}(\Delta^{2})]\\ & \;+2k\pi^{⊤}(H\Sigma (\beta {)}^{-1}H^{⊤}{)}^{-1}H\Sigma (\beta {)}^{-1}W\Sigma (\beta {)}^{-1}H^{⊤}\\ & \;\times (H\Sigma (\beta {)}^{-1}H^{⊤}{)}^{-1}\pi E[\chi_{q+2}^{-2}(\Delta^{2})]\\ & =R_{1}({\hat{\beta }}_{n}^{pl};W)-k\text{tr}({𝐀}_{11})\{2E[\chi_{q+2}^{-2}(\Delta^{2})]-kE[\chi_{q+2}^{-4}(\Delta^{2})]\}\\ & \;+k(k+4)(\eta_{1}^{⊤}{𝐀}_{11}\eta_{1})E[\chi_{q+4}^{-4}(\Delta^{2})],\;\text{by equation 17}. \end{array}$$ (18)

Finally, we consider the risk expression for ${\hat{\beta }}_{n}^{S+}$:


$$\begin{array}{cc} R_{5}({\hat{\beta }}_{n}^{S+};W) & =\lim_{n\to \infty }E[n({\hat{\beta }}_{n}^{S+}-\beta {)}^{⊤}W({\hat{\beta }}_{n}^{S+}-\beta )]\\ & =\lim_{n\to \infty }E[n\{({\hat{\beta }}_{n}^{S}-\beta )-(1-k_{n}{𝒲}_{n}^{-1})I({𝒲}_{n}<k_{n})({\hat{\beta }}_{n}^{pl}-{\hat{\beta }}_{n}^{R}){\}}^{⊤}W\\ & \;\times \{({\hat{\beta }}_{n}^{S}-\beta )-(1-k_{n}{𝒲}_{n}^{-1})I({𝒲}_{n}<k_{n})({\hat{\beta }}_{n}^{pl}-{\hat{\beta }}_{n}^{R})\}]\\ & =\lim_{n\to \infty }E[n({\hat{\beta }}_{n}^{S}-\beta {)}^{⊤}W({\hat{\beta }}_{n}^{S}-\beta )]\\ & \;+\lim_{n\to \infty }E[(1-k_{n}{𝒲}_{n}^{-1}{)}^{2}I({𝒲}_{n}<k_{n})n({\hat{\beta }}_{n}^{pl}-{\hat{\beta }}_{n}^{R}{)}^{⊤}W({\hat{\beta }}_{n}^{pl}-{\hat{\beta }}_{n}^{R})]\\ & \;-2\lim_{n\to \infty }E[(1-k_{n}{𝒲}_{n}^{-1})I({𝒲}_{n}<k_{n})n({\hat{\beta }}_{n}^{S}-\beta {)}^{⊤}W({\hat{\beta }}_{n}^{pl}-{\hat{\beta }}_{n}^{R})]\\ & =R_{4}({\hat{\beta }}_{n}^{S},𝐖)+\lim E[(1-k_{n}{𝒲}_{n}^{-1}{)}^{2}I({𝒲}_{n}<k_{n})n({\hat{\beta }}_{n}^{pl}-{\hat{\beta }}_{n}^{R}{)}^{\prime }𝐖({\hat{\beta }}_{n}^{pl}-{\hat{\beta }}_{n}^{R})]\\ & \;-2\lim_{n\to \infty }E[(1-k_{n}{𝒲}_{n}^{-1})I({𝒲}_{n}<k_{n})\{n({\hat{\beta }}_{n}^{pl}-\beta )\\ & \;+(1-k_{n}{𝒲}_{n}^{-1})n({\hat{\beta }}_{n}^{pl}-{\hat{\beta }}_{n}^{R})-n({\hat{\beta }}_{n}^{pl}-{\hat{\beta }}_{n}^{R})\}𝐖({\hat{\beta }}_{n}^{pl}-{\hat{\beta }}_{n}^{R})]\\ & =R_{4}({\hat{\beta }}_{n}^{S};𝐖)-\lim_{n\to \infty }E[(1-k_{n}{𝒲}_{n}^{-1}{)}^{2}I({𝒲}_{n}<k)]\{n({\hat{\beta }}_{n}^{pl}-{\hat{\beta }}_{n}^{R}{)}^{⊤}𝐖({\hat{\beta }}_{n}^{pl}-{\hat{\beta }}_{n}^{R})]\\ & \;-2\lim E\{[n({\hat{\beta }}_{n}^{pl}-{\hat{\beta }}_{n}^{R}{)}^{⊤}𝐖({\hat{\beta }}_{n}^{pl}-{\hat{\beta }}_{n}^{R})](1-k_{n}{𝒲}_{n}^{-1})I({𝒲}_{n}<k_{n})\}\\ & \;+2\lim E\{[n({\hat{\beta }}_{n}^{pl}-{\hat{\beta }}_{n}^{R}{)}^{⊤}𝐖({\hat{\beta }}_{n}^{pl}-{\hat{\beta }}_{n}^{R})](1-k_{n}{𝒲}_{n}^{-1})I({𝒲}_{n}<k_{n})\}\\ & =R_{4}({\hat{\beta }}_{n}^{S};W)-\text{tr}(A_{11})E[(1-k\chi_{q+2}^{-2}(\Delta^{2}){)}^{2}I(\chi_{q+2}^{2}(\Delta^{2})<k)]\\ & \;-(\eta_{1}^{⊤}A_{11}\eta_{1})\{2E[(1-k\chi_{q+2}^{-2}(\Delta^{2}))I(\chi_{q+2}^{2}(\Delta^{2})<k)]\\ & \;-E[(1-k\chi_{q+4}^{-2}(\Delta^{2}){)}^{2}I(\chi_{q+4}^{2}(\Delta^{2})<k)]\}. \end{array}$$

## References

[1] Cox DR. Regression Models and Life-Tables. Journal of the Royal Statistical Society Series B: Statistical Methodology. 1972;34(2):187–202. doi: 10.1111/j.2517-6161.1972.tb00899.x

[2] Andersen PK, Gill RD. Cox’s Regression Model for Counting Processes: A Large Sample Study. Ann Statist. 1982;10(4). doi: 10.1214/aos/1176345976

[3] Fleming RT. Counting processes and survival analysis. John Wiley & Sons. 2005. 10.1002/9781118150672

[4] Andersen PK, Borgan O, Gill RD, Keiding N. Statistical models based on counting processes. Springer New York. 1995. 10.1007/978-1-4612-4348-9

[5] Stein C. Inadmissibility of the usual estimator for the mean of a multivariate normal distribution. Contribution to the Theory of Statistics. University of California Press. 1956. 197–206. 10.1525/9780520313880-018

[6] James W, Stein C. Estimation with Quadratic Loss. Springer Series in Statistics. Springer New York. 1992. 443–60. 10.1007/978-1-4612-0919-5_30

[7] Ahmed SE, Arabi Belaghi R, Hussein AA. Efficient Post-Shrinkage Estimation Strategies in High-Dimensional Cox’s Proportional Hazards Models. Entropy (Basel). 2025;27(3):254. doi: 10.3390/e27030254 40149178 PMC11941331

[8] Zareamoghaddam H, Ahmed SE, Provost SB. Shrinkage estimation applied to a semi-nonparametric regression model. Int J Biostat. 2020;17(1):23–38. doi: 10.1515/ijb-2018-0109 32769222

[9] Arashi M, Norouzirad M. Advances in shrinkage and penalized estimation strategies: Honoring the contributions of Professor A K Md. Ehsanes Saleh. Springer. 2025. 10.1007/978-3-031-94050-7

[10] Shih J-H, Lin T-Y, Jimichi M, Emura T. Robust ridge M-estimators with pretest and Stein-rule shrinkage for an intercept term. Jpn J Stat Data Sci. 2020;4(1):107–50. doi: 10.1007/s42081-020-00089-6

[11] Raheem E, Ahmed SE, Liu S. Stein-rule M-estimation in sparse partially linear models. Jpn J Stat Data Sci. 2023;7(1):507–35. doi: 10.1007/s42081-023-00231-0

[12] Efron B. Machine learning and the James–Stein estimator. Jpn J Stat Data Sci. 2023;7(1):257–66. doi: 10.1007/s42081-023-00209-y

[13] Hossain S, Ahmed SE. Penalized and Shrinkage Estimation in the Cox Proportional Hazards Model. Communications in Statistics - Theory and Methods. 2014;43(5):1026–40. doi: 10.1080/03610926.2013.826368

[14] Tibshirani R. The lasso method for variable selection in the Cox model. Stat Med. 1997;16(4):385–95. doi: 10.1002/(sici)1097-0258(19970228)16:4<385::aid-sim380>3.0.co;2-3 9044528

[15] Ng CT, Yu CW. Modified SCAD penalty for constrained variable selection problems. Statistical Methodology. 2014;21:109–34. doi: 10.1016/j.stamet.2014.05.001

[16] Utazirubanda JC, Leon T, Ngom P. Variable selection with Group LASSO approach: Application to Cox regression with frailty model. Commun Stat Simul Comput. 2021;50(3):881–901. doi: 10.1080/03610918.2019.1571605 34248255 PMC8261624

[17] Elashoff R, Li N. Joint Modeling of Longitudinal and Time-to-Event Data. Chapman and Hall/CRC. 2016. 10.1201/978131537487

[18] Efron B. The Efficiency of Cox’s Likelihood Function for Censored Data. Journal of the American Statistical Association. 1977;72(359):557–65. doi: 10.1080/01621459.1977.10480613

[19] Kalbfleisch JD, Prentice RL. The statistical analysis of failure time data. John Wiley & Sons. 2002. 10.1002/9781118032985

[20] van der Vaart AW. Asymptotic statistics. Cambridge University Press. 1998. 10.1017/CBO9780511802256

[21] Zou H. The Adaptive Lasso and Its Oracle Properties. Journal of the American Statistical Association. 2006;101(476):1418–29. doi: 10.1198/016214506000000735

[22] Zou H, Hastie T. Regularization and Variable Selection Via the Elastic Net. Journal of the Royal Statistical Society Series B: Statistical Methodology. 2005;67(2):301–20. doi: 10.1111/j.1467-9868.2005.00503.x

[23] Suissa S. Immortal Time Bias in Pharmacoepidemiology. American Journal of Epidemiology. 2008;167(4):492–9. doi: 10.1093/aje/kwm324 18056625

[24] Therneau TM, Lumley T. Package ‘survival’. R Top Doc. 2015;128(10):28–33.

